# Prediction of muscle loss after stroke by analysis of corticospinal tract

**DOI:** 10.1515/tnsci-2020-0114

**Published:** 2020-09-09

**Authors:** Ah Young Lee, Kyu Tae Choi, Min Cheol Chang

**Affiliations:** Department of Rehabilitation Medicine, Daegu Veterans Health Service Medical Center, Daegu, Republic of Korea; Department of Physical Medicine and Rehabilitation, College of Medicine, Yeungnam University, 317-1, Daemyungdong, Namku, Daegu, 705-717, Republic of Korea

**Keywords:** corticospinal tract, diffusion tensor tractography, muscle loss, prediction, stroke

## Abstract

**Introduction:**

Skeletal muscle loss induces a poor rehabilitation outcome after stroke. Little is known about the usefulness of diffusion tensor tractography (DTT) findings of the corticospinal tract (CST) in terms of predicting muscle loss in affected limbs after stroke.

**Methods:**

This research was designed as a preliminary study. Forty-four patients, with stroke onset more than one year earlier, were recruited. DTT was performed within 7–30 days after stroke onset. The patients were classified into two groups based on the DTT findings: a DTT+ group, in which the CST was preserved, and a DTT− group, in which the CST was interrupted by the stroke lesion. Additionally, the patients’ functions were evaluated based on the modified Brunnstrom classification and functional ambulation category.

**Results:**

In the DTT− group, the values of the lean tissue mass of the affected upper and lower limbs were smaller than those of the unaffected side. On the other hand, in the DTT+ group, the values of the lean tissue mass between the affected and unaffected limbs were not significantly different.

**Conclusion:**

The DTT evaluation of CST at the early stage of stroke may be useful for predicting muscle loss of the affected limb at the chronic stage in stroke patients.

## 1 Introduction

Stroke is one of the leading neurological disorders causing functional impairment. Stroke patients with moderate to severe disability cannot ambulate independently and have difficulty in performing activities of daily living. Inactivity or minimal use of muscles is known to cause a decrease in the muscle mass of the affected limbs [1]. A decrease in skeletal muscle mass results in weakness, fatigue, and poor motor function rehabilitation outcomes, such as walking and performing activities of daily living [2]. Therefore, prediction of loss of muscle mass is important to elucidate the appropriate rehabilitation strategy in patients with stroke.

Diffusion tensor tractography (DTT) is a technique that can visualize the architecture and integrity of the neural tracts in the brain [3]. The integrity of neural tracts after stroke is closely related to the outcome of rehabilitation treatment [3,4]. In several previous studies, the usefulness of DTT for predicting outcomes of several functions, such as motor, sensory, speech, and cognition, was demonstrated [3,4]. However, to date, no study has evaluated the loss of muscle mass according to the DTT findings.

The corticospinal tract (CST) is the most important neural tract for motor function in humans [5]. In several previous studies, the preserved integrity of CST after stroke is reported to be one of the most important factors for the recovery of motor function in stroke patients [3,5,6]. Therefore, knowledge of the state of the CST would be helpful to predict the functional ability of stroke patients. Limb disuse after stroke-induced functional disability reduces the muscle mass in patients with stroke [1,2]. Accordingly, it could be hypothesized that evaluation of the CST status by means of DTT may be helpful for predicting the loss of muscle mass in stroke patients. Previously, Kwak et al. reported that the CSTs of elderly people with sarcopenia were deteriorated and that the CST status was correlated with the handgrip strength [7].

In the current study, we depicted the CST in stroke patients by using DTT and evaluated the change in the muscle mass of the affected limbs in stroke patients according to the state of the CST on DTT.

## 2 Methods

### 2.1 Subjects

This research was a cross-sectionally designed preliminary study. During January 2019, we recruited 42 patients (21 men, 21 women; 58.9 ± 10.8 years; range, 39–77 years; 18 cerebral infarcts, 24 intracerebral hemorrhages; 29.6 ± 24.6 months after stroke onset) from the outpatient rehabilitation clinic of our university hospital. The following inclusion criteria were applied: (1) a history of stroke, (2) ≥1 year after stroke onset, (3) age between 21 and 79 years, (4) hemiplegia after stroke, (5) DTT performed within 7–30 days of onset, and (6) absence of serious medical complications, such as pneumonia or cardiac problems, from onset to final evaluation.


**Informed consent:** All patients gave written informed consent.
**Ethical approval:** The study has been complied with all the relevant national regulations, institutional policies and in accordance with the tenets of the Declaration of Helsinki. Our study protocol was approved by the Institutional Review Board of our university hospital.

### 2.2 DTT

We used the DTT data obtained previously at the subacute stage after stroke. The mean duration from onset to diffusion tensor imaging (DTI) was 15.3 ± 5.3 days (range: 8–29 days). DTI was performed using a sensitivity-encoding head coil on a 1.5-T Philips Gyroscan Intera (Hoffman-LaRoche Ltd, Best, the Netherlands) with single-shot echo-planar imaging and navigator echo. Sixty contiguous slices (matrix = 128 × 128, field-of-view = 221 × 221 mm^2^, TE = 76 mm, TR = 10,726 ms, SENSE factor = 2; EPI factor = 67 and *b* = 1,000 s/mm/mm, NEX = 1, with a 2.3-mm slice thickness) were acquired for each of the 32 noncollinear diffusion-sensitizing gradients. The scanning time for DTI acquisition was 7 min 32 s. Head motion effects and image distortions due to eddy currents were corrected by affine multiscale two-dimensional (2D) registration.

**Figure 1:**
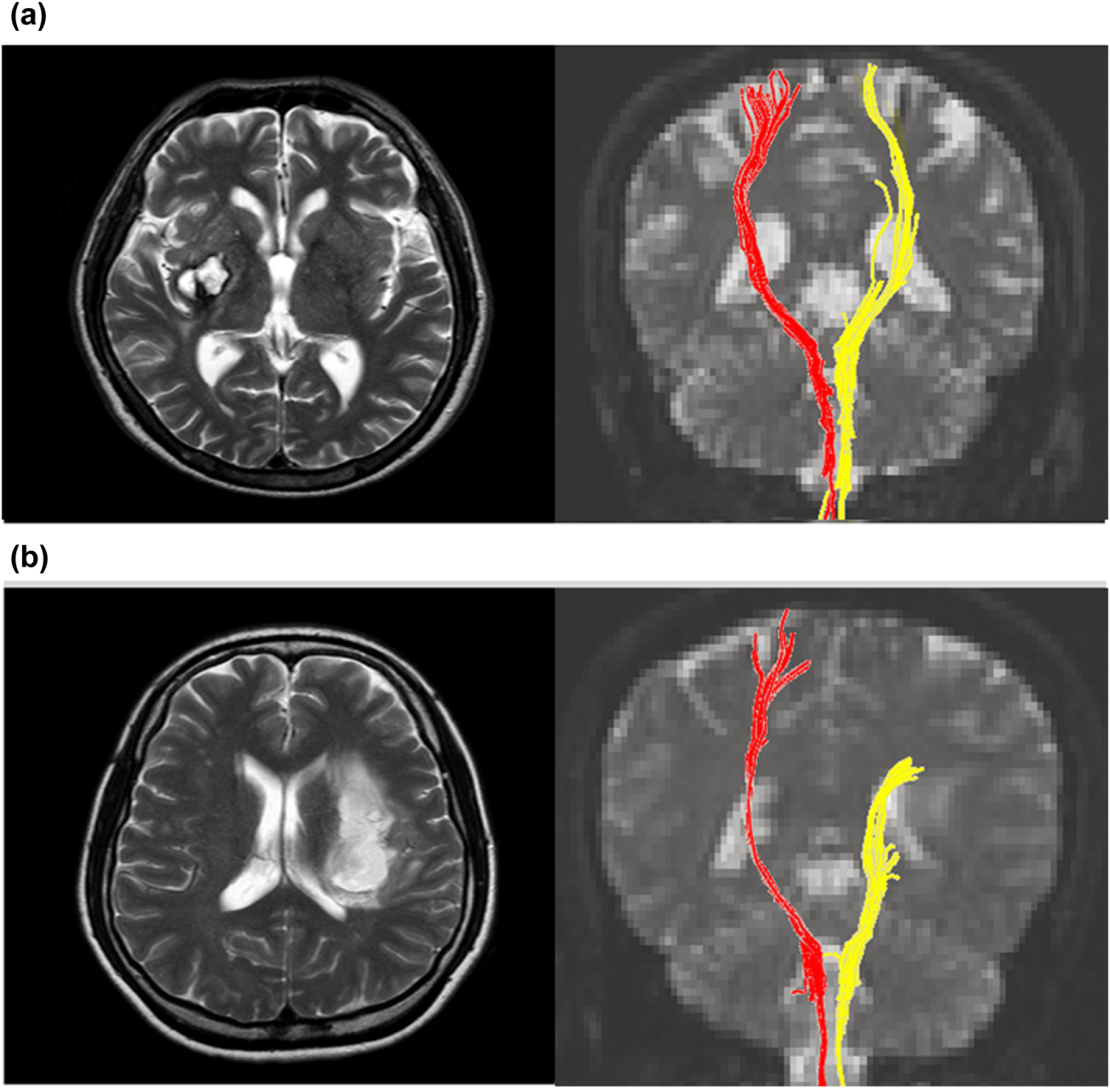
Classification according to the state of the CST on DTT. Axial *T*2-weighted magnetic resonance images (left) and coronal DTT images (right). (a) DTT+: the CST was preserved around the infarct. (b) DTT−: the CST was interrupted by the stroke lesion.

The integrity of the CST was evaluated using Fiber Assignment by Continuous Tracking, a three-dimensional fiber reconstruction algorithm in the Philips PRIDE software (Philips Medical Systems, Best, The Netherlands) [3]. CST fiber tracking was performed using a fractional anisotropy (FA) threshold of *>*0.2 and a direction threshold of 60°. In each case, a seed region-of-interest (ROI) was drawn in the CST portion of the anterior mid-pons on a 2D FA color map, and another ROI was drawn in the CST portion of the anterior low-pons on a 2D FA color map. Fiber tracts passing through both ROIs were designated as the final tracts of interest (Figure 1). We divided the included patients into two groups according to the DTT findings: DTT+ group, in which the CST was preserved around the infarct; DTT− group, in which the CST was interrupted by the infarct.

### 2.3 Body composition

On the day of recruitment, dual energy X-ray absorptiometry (Discovery, Hologic Inc., Marlborough, MA) was performed to analyze body composition. All patients were asked to wear a gown and remove all jewelry and other materials that could interfere with the result of the examination. Dual energy X-ray absorptiometry measures lean tissue, which includes lean muscle and connective tissue, and is an accurate proxy measure of muscle mass [8]. Lean tissue mass was measured in bilateral upper and lower limbs. The results of the examinations were reviewed and analyzed by a nuclear medicine physician. The regions of interest were designated as follows: the lean tissue in the upper limb was measured from the lower edge of the distal phalanx of the middle finger to the upper edge of the acromion, and the medial and lateral boundaries were placed to encompasses all soft tissues; lean tissue in the lower limb was measured from the lower edge of the ischium to the bottom edge of the distal phalanx of the hallux, and the medial and lateral boundaries were drawn to encompass all soft tissues.

### 2.4 Evaluation of the motor function

On the day of recruitment, the function of the affected hand was categorized using the modified Brunnstrom classification (MBC) [3], as follows: (1) unable to move fingers voluntarily; (2) able to move fingers voluntarily; (3) able to close the affected hand voluntarily but unable to open the hand; (4) able to grasp a card between thumb and the medial side of the index finger and able to extend fingers slightly; (5) able to pick up and hold a glass and extend fingers; and (6) able to catch and throw a ball in a near-normal fashion and able to button and unbutton a shirt. Walking ability was quantified using the standardized functional ambulation category (FAC) [3], which is based on the characterization of the level of assistance required during a 15-min walk. The six FACs are as follows: (1) nonambulatory; (2) a need for continuous support from one person; (3) a need for intermittent support from one person; (4) requirement for verbal supervision only; (5) help required on stairs and uneven surfaces; and (6) can walk independently anywhere.

### 2.5 Statistical analysis

Statistical analysis was performed using SPSS 23.0 software (SPSS INC, Chicago, IL, USA). The statistical significance of differences in the lean tissue mass between the affected and unaffected sides in each group was analyzed using the Mann–Whitney test. The differences in lean tissue mass between the affected and unaffected sides in total patients were also compared with the Mann–Whitney test. Furthermore, the differences in demographic data, MBC, and the FAC between DTT+ and DTT− groups were determined using the Mann–Whitney test and the chi-squared test. Statistical significance was accepted at *p* values of <0.05.

## 3 Results

According to the DTT findings, 22 and 20 patients were allocated to the DTT+ and DTT− groups, respectively. There was no significant difference in demographic parameters between the DTT+ and DTT− groups (Table 1). In the DTT− group, the values of the lean tissue mass of the upper and lower limbs of the affected side were smaller than those of the unaffected side (Table 2). In contrast, in the DTT+ group, the values of the lean tissue mass between the affected and unaffected upper limbs were not statistically significantly different (Table 2). Additionally, those of the lower limbs were not significantly different between the affected and unaffected sides.

**Table 1:** Demographic data for patients in the DTT+ group and DTT− group

	DTT+	DTT−	*p*
Demographic data			
Number of patients, *n*	22	20	
Age, years	61.5 ± 10.8	56.0 ± 10.4	0.127
Male:female, *n*	11:11	10:10	1.000
Height, cm	165.3 ± 11.3	166.3 ± 7.4	0.960
Weight, kg	68.8 ± 10.8	71.1 ± 8.4	0.464
Body mass index, kg/m^2^	25.2 ± 3.0	25.8 ± 3.1	0.614
Months from onset	29.7 ± 24.9	29.5 ± 24.9	0.940
Lesion type (infarct/hemorrhage), *n*	11/11	7/13	0.327
Lesion side (right/left), *n*	13/9	10/10	0.554
Days to DTT	16.1 ± 5.1	14.3 ± 5.4	0.250

DTT: diffusion tensor tractography.

DTT+ group: CST was preserved around the infarct; DTT− group: CST was interrupted by the infarct.

Values are presented as mean ± standard deviation.

**Table 2:** Comparison of the lean tissue mass between the affected and unaffected limbs

	Affected	Unaffected	*p*
DTT+ group (g)			
Upper limb	2333.0 ± 596.9	2409.1 ± 687.2	0.869
Lower limb	6899.7 ± 1756.7	6934.0 ± 1736.2	0.934
DTT− group (g)			
Upper limb	2379.4 ± 537.0	2723.0 ± 538.9	0.038
Lower limb	5813.5 ± 1527.5	6714.9 ± 1757.8	0.043
Total patients (g)			
Upper limb	2355.1 ± 562.7	2558.6 ± 633.8	0.138
Lower limb	6382.5 ± 1721.5	6829.7 ± 1728.6	0.181

DTT: diffusion tensor tractography.

DTT+ group: CST was preserved around the infarct; DTT− group: CST was interrupted by the infarct.

Values are presented as mean ± standard deviation.

The MBC and FAC scores in each patient are presented in Table 3. In the intergroup comparison of motor function, the MBC and FAC in the DTT+ group were significantly larger than those in the DTT− group (MBC: DTT+ group = 5.6 ± 0.6, DTT− group = 2.5 ± 1.1, *p* < 0.001; FAC: DTT+ group = 3.8 ± 0.7, DTT− group = 2.5 ± 0.8, *p* < 0.001).

**Table 3:** MBC and FAC scores of each patient

	MBC	FAC
DTT+ group		
1	5	3
2	6	5
3	6	4
4	5	4
5	6	4
6	5	3
7	6	4
8	5	3
9	6	3
10	6	3
11	5	3
12	5	4
13	6	4
14	6	4
15	6	5
16	4	4
17	6	5
18	6	4
19	6	3
20	6	3
21	6	4
22	5	4
DTT− group		
1	1	2
2	3	2
3	3	2
4	3	2
5	1	1
6	3	3
7	3	3
8	2	4
9	2	3
10	4	3
11	4	3
12	2	3
13	2	2
14	2	2
15	1	1
16	1	4
17	5	3
18	2	2
19	3	3
20	2	2

## 4 Discussion

In this study, we investigated whether the DTT findings of the CST during the early stage of stroke can predict loss of muscle mass in the affected limbs at the chronic stage of stroke. We classified patients into two groups based on the CST integrity as observed on DTT. In patients whose CST was interrupted, muscle mass of the affected upper and lower limbs was found to be significantly smaller than those of the affected side. However, in the patients whose CST was preserved, muscle mass of the affected upper and lower limbs was not significantly reduced. Our results were consistent with the findings of previous studies that showed that muscle mass in the paretic limb was reduced and that this reduction was associated with functional disability after stroke [2,8,9,10]. In our study, the DTT− group (with an interrupted CST) showed less functional ability than the DTT+ group (with a preserved CST). Furthermore, the average MBC and FAC of the patients in the DTT− group were 2.5 and 2.5, respectively. Considering that an MBC ≥ 5 and a FAC ≥ 3 are the cut-off values for voluntary hand movement and gait achievement [3,5], the DTT− group patients’ affected limbs were nonfunctional, which seems to have resulted in disuse atrophy of the muscle mass of the paretic limbs. The decrease in central activation following interruption of the CST also appears to have contributed to the decrease in the muscle mass of the affected limbs [1,2]. In contrast, patients who had preserved CST had, on average, an MBC of 5.6 and a FAC of 3.8, which indicated that they were able to use their affected limbs functionally, preventing muscle atrophy of the affected limbs.

Several DTT studies have demonstrated the usefulness of DTT for predicting the outcome of motor function [3,5,6]. However, to date, its ability to predict muscle mass reduction has not been studied. Although previous studies reported that muscle loss after stroke contributes to a poor rehabilitation outcome after stroke [2,8], clinicians frequently do not consider the possibility of muscle wasting after stroke. Rehabilitative management, including an exercise program for preventing muscle loss in patients with a high risk of skeletal muscle loss (CST interrupted on DTT), would enhance functional outcome in stroke patients.

In conclusion, we showed that DTT findings of the CST during the early stage of stroke may predict the occurrence of muscle loss in the affected limbs. The prediction of muscle loss in stroke patients using DTT could be applicable only to patients whose CST was interrupted. No previous study had investigated the usefulness of DTT analysis for predicting muscle wasting after stroke. For patients whose CST was interrupted according to DTT performed in the early stage of stroke, active treatment for preventing or restricting muscle loss is necessary. The present study had some limitations. First, the number of included patients was small. Second, we did not perform a longitudinal study. Third, we did not adjust confounding factors that may effect changes in muscle mass, such as age, spasticity, and nutrition status. Further studies that address these limitations are warranted in future.
